# Onwards and upwards: *European Journal of Medical Research *continues as an open access publication

**DOI:** 10.1186/2047-783X-17-1

**Published:** 2012-01-30

**Authors:** Stefan Busch, Dieter Häussinger

**Affiliations:** 1 BioMed Central, 236 Gray's Inn Road, London, WC1X 8HB, UK; 2 University Hospital Düsseldorf, Department of Gastroenterology, Hepatology and Infectious Diseases, Heinrich Heine University, 40225 Düsseldorf, Germany

## Abstract

The well-established *European Journal of Medical Research *has joined BioMed Central's portfolio of journals in January 2012, converting to the open access publishing model. Since its launch in 1995 the journal has been a print-only publication; from now on, it continues as an open access, online-only journal. The conversion to open access opens up the potential for the journal to become a leading, globally visible title in the field of general medicine over the coming years.

## Editorial

As of today, at the outset of its 17^th ^volume, the *European Journal of Medical Research *is an open access publication, having joined BioMed Central's portfolio of journals. The change in the publishing model awards this well-established title with an opportunity to develop into a leading general medicine journal over the coming years.

Founded by the Munich-based publishing house Holzapfel Verlag in 1995, the journal soon became a respected and broadly indexed resource. Until the end of 2011, however, the journal remained a print-only publication. While many other aspects of the journal will continue without change, the publishing 'format' is now changing, from print-only to online-only and from subscription-based economics to the open access model with article processing charges borne, after peer review and upon acceptance, by authors' home institutions or research funders. The rapid growth of high-quality open access publications is rooted in the fact that an increasing number of universities, funding agencies and governmental agencies support or even mandate open access, in different ways and through different means.

The journal's new publisher, BioMed Central (Springer's imprint for open access journals in biology and medical disciplines) has over recent years accumulated a wealth of experience with journal transfers and conversions to open access. A rapidly growing number of journals in BioMed Central's portfolio moved there from other publishing arrangements. In such cases, a clear trend has emerged that shows an increase of these journals' impact factors after two to three years when the open access articles start forming the basis of the calculations. Higher visibility and unrestricted access lead to increased impact [1], of which the impact factor is only one aspect. Taken as a proxy, however, it serves as an indicator of the effect of open access on visibility and usage. Cases in point are *Acta Veterinaria Scandinavica*, which has seen its impact factor quadruple since its move to BioMed Central in 2006, and *Journal of Cardiovascular Magnetic Resonance*, another long-established society publication, which has seen its impact factor almost treble to 4.3 three years after it became an open access title [2].

We are optimistic that we can achieve a similar effect for *European Journal of Medical Research*, and the Editorial Board and BioMed Central as the publisher will work together to achieve this. As a first step towards this aim, the new publisher has converted the technical format of all articles published in the journal in 2010 and 2011 to make them freely available for a global readership.

While the scope of the journal remains unchanged, with continued focus on clinical research, there is from now on unrestricted 'space' to publish articles from across the full spectrum of medical disciplines, with no restrictions set by page budgets. With regard to acceptability of research that falls into the journal's scope, the only restricting factor is quality, and the guardians of quality are the members of the Editorial Board. Future changes of the size and composition of the board will reflect a number of factors, such as the overall growth of the journal, the 'share' of articles from the diversity of medical subjects, and the objective to build a truly pan-European base for the journal.

The journal's success story since its launch 17 years ago is the achievement of its founders and editors, Ferdinand Holzapfel, Monika Ortner-Bach, and Professor Nepomuk Zöllner as the first Editor-in-Chief, together with so many colleagues who contributed and continue to contribute as authors, editors, and reviewers. The future of *European Journal of Medical Research *lies in open access. What further changes will we see as a result of its re-launch as an open access journal? Which new audiences will it find, and which medical disciplines will be most represented among the publications? We are curious to find out over the coming years, and this curiosity is based on our confidence that the conversion to open access and online publishing puts the journal in a strong position to continue its success story. We look forward to work with 'old' and 'new' colleagues on this new chapter in the story entitled '*European Journal of Medical Research*'.
